# Design and validation of a novel 3D-printed glenohumeral fusion prosthesis for the reconstruction of proximal humerus bone defects: a biomechanical study

**DOI:** 10.3389/fbioe.2024.1428446

**Published:** 2024-07-08

**Authors:** Jiaming Lin, Guohui Song, Anfei Huang, Jinxin Hu, Qinglian Tang, Jinchang Lu, Yufeng Huang, Ming Gong, Xiaojun Zhu, Jin Wang

**Affiliations:** Department of Musculoskeletal Oncology, State Key Laboratory of Oncology in South China, Guangdong Provincial Clinical Research Center for Cancer, Sun Yat-sen University Cancer Center, Guangzhou, China

**Keywords:** proximal humerus tumor, proximal humerus bone defect, glenohumeral fusion, 3D-printed glenohumeral fusion prosthesis, biomechanics

## Abstract

**Background:**

All available methods for reconstruction after proximal humerus tumor resection have disadvantages, and the optimal reconstruction method remains uncertain. This study aimed to design a novel 3D-printed glenohumeral fusion prosthesis and verify its feasibility and safety using biomechanical methods.

**Methods:**

We verified the feasibility and safety of the 3D-printed glenohumeral fusion prosthesis by finite element analysis and biomechanical experimentation. In the finite element analysis, three reconstruction methods were used, and displacement and von Mises stress were observed; on this basis, in the biomechanical experiment, models constructed with sawbones were classified into two groups. The force‒displacement curve of the 3D-printed prosthesis was evaluated.

**Results:**

In terms of displacement, the finite element analysis showed greater overall stability for the novel prosthesis than traditional glenohumeral joint arthrodesis. There was no obvious stress concentration in the internal part of the 3D-printed glenohumeral fusion prosthesis; the stable structure bore most of the stress, and the force was well distributed. Adding lateral plate fixation improved the stability and mechanical properties of the prosthesis. Furthermore, the biomechanical results showed that without lateral plate fixation, the total displacement of the prosthesis doubled; adding lateral plate fixation could reduce and disperse strain on the glenoid.

**Conclusion:**

The design of the 3D-printed glenohumeral fusion prosthesis was rational, and its stability and mechanical properties were better than those of traditional glenohumeral joint arthrodesis. Biomechanical verification demonstrated the feasibility and safety of this prosthesis, indicating its potential for proximal humerus bone defect reconstruction after tumor resection.

## 1 Introduction

The shoulder girdle is the third most common site of bone tumors, and the proximal humerus is the most commonly affected component (Wittig et al., 2001). Methods for reconstruction after the resection of malignant proximal humerus tumors include autologous bone grafting, osteoarticular allografting, the application of allograft-prosthesis composites, artificial prosthesis placement, and arthrodesis with an intercalary allograft and a vascularized fibular graft (Abdeen et al., 2009; Teunis et al., 2014; Sirveaux, 2019). The above existing reconstruction methods can be divided into two types, namely, glenohumeral fusion reconstruction and glenohumeral nonfusion reconstruction. Achieving stability in the shoulder is difficult with glenohumeral nonfusion reconstruction (Liu et al., 2014; Tang et al., 2015; Yang et al., 2021), which also makes it difficult to achieve good shoulder function due to defects of the deltoid or axillary nerve (Wang et al., 2011; Wang et al., 2015; Yang et al., 2021). However, glenohumeral fusion reconstruction restores stability of the shoulder joint, and a stable and painless shoulder joint is an essential prerequisite for upper limb function. In addition, after glenohumeral fusion reconstruction, the motion of the scapula can partially compensate for the motion of the glenohumeral joint, restoring the range of motion of the shoulder joint to a certain extent and qualifying the approach as an excellent reconstruction technique. The traditional method for glenohumeral fusion reconstruction is arthrodesis with allografts and vascularized fibular grafts. There are many drawbacks to this method, including a long operation time, great trauma, a long time required for fusion between the allograft and host bone, rejection reactions, bone resorption, and bone nonunion, which may lead to additional related complications and ultimately reconstruction failure (Fuchs et al., 2005; Wang et al., 2011; Bilgin, 2012; Wieser et al., 2013; Padiolleau et al., 2014; Mimata et al., 2015). The so-called glenohumeral fusion prosthesis made by traditional techniques relies on only a few screws to achieve stability in the initial stage of reconstruction, but this approach does not allow long-term biological stability to be achieved through integration at the interface between the bone and prosthesis (O’Connor et al., 1996). As a result, the failure rate of these traditional prostheses is very high in the long term (O’Connor et al., 1996). Facing the drawbacks of the above two methods of glenohumeral fusion reconstruction, we applied 3D printing technology for arthrodesis of the glenohumeral joint, which will hopefully solve this clinical problem.

We designed a novel 3D-printed glenohumeral fusion prosthesis, verified its feasibility and safety, and explored the potential improvement in biomechanical stability through a lateral plate fixation with it by finite element analysis and biomechanical experiments, providing mechanical evidence for its clinical application.

## 2 Materials and methods

### 2.1 Design of the 3D-printed glenohumeral fusion prosthesis

**FIGURE 1:**
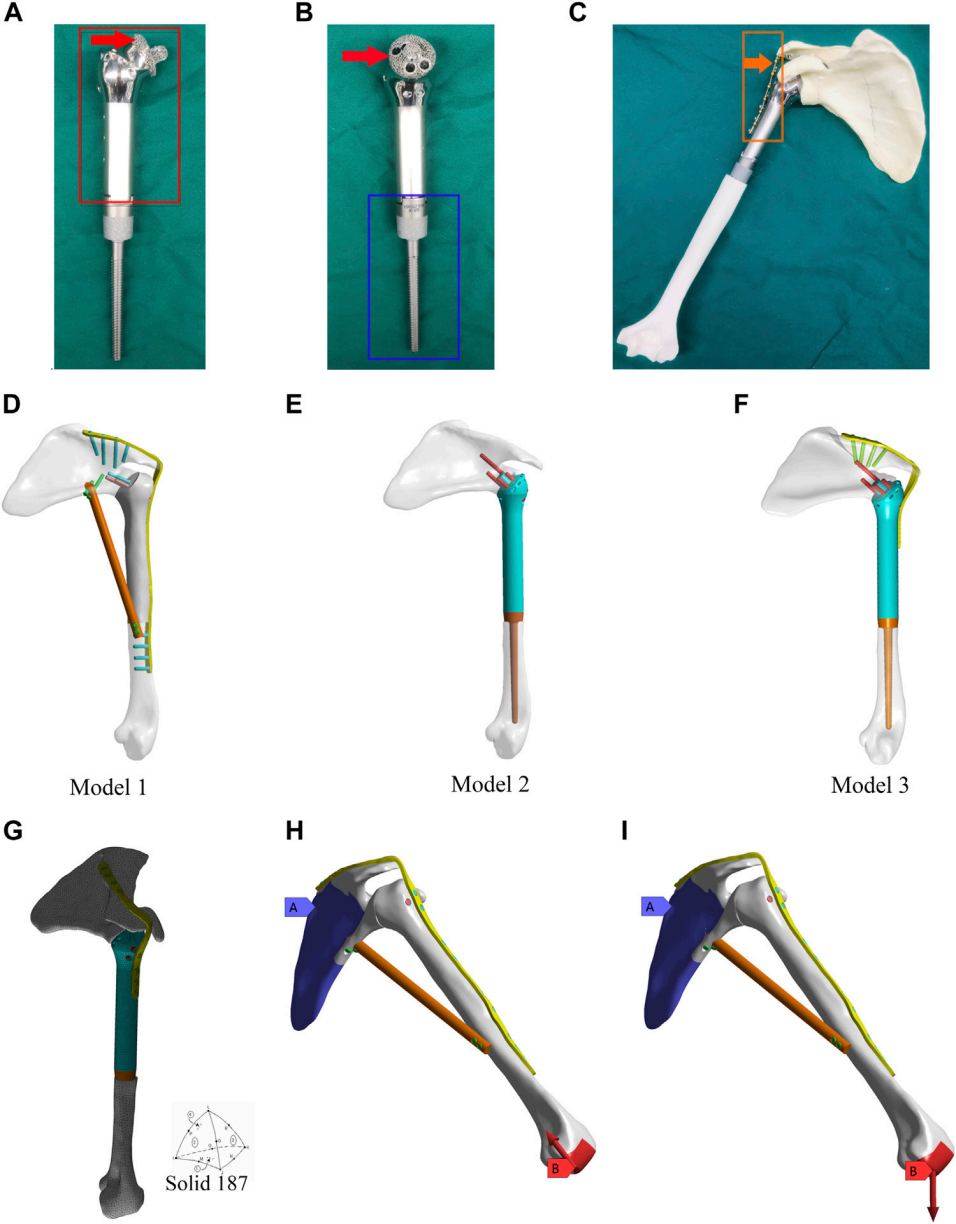
Design of the 3D-printed glenohumeral fusion prosthesis and model construction, and element division method and loading modes in the finite element analysis. **(A,B)** Design details of the 3D-printed glenohumeral fusion prosthesis. **(C)** Biomimetic bones (sawbones) of the shoulder joint were used to construct biomechanical experimental models for simulating proximal humerus bone defect reconstruction after proximal humerus tumor resection with the 3D-printed glenohumeral fusion prosthesis. **(D–F)** Three reconstruction models were created, all of which simulated the reconstruction of the bone defect after a 15-cm osteotomy in the proximal humerus. **(D)** Model 1 represented traditional glenohumeral arthrodesis with an intercalary allograft and a vascularized fibular graft. **(E)** Model 2 represented the 3D-printed glenohumeral fusion prosthesis. **(F)** Model 3 represented the 3D-printed glenohumeral fusion prosthesis with a metal plate fixed on the spine of the scapula. **(G)** The contact was set, and the grid was divided freely with solid 187 as the solid unit and 3 mm as the unit size. **(H,I)** The scapula was bound and fixed, and two loading modes were applied to the distal end of the humerus. **(H)** The first loading mode consisted of an axial load of 700 N along the humeral axis, which was equivalent to the pressure on the glenohumeral joint during arm elevation (Model 1 is taken as an example to illustrate two loading modes). **(I)** The loading mode consisted of a vertical downward load of 42.532 N, which was equivalent to the weight of the upper limb.

The design of the novel prosthesis mimics the technical characteristics of traditional glenohumeral joint arthrodesis (O’Connor et al., 1996; Wang et al., 2011). To achieve the purpose of fusion, design details were established for “initial stability (mechanical fusion)” and “long-term stability (biological fusion)” (Figures 1C, F). The prosthesis consists of three parts, including the main part of the prosthesis (Figure 1A, shown in the red box), the intramedullary stem (Figure 1B, shown in the blue box), and the fixation plate (Figure 1C, shown in the orange box).

The initial stability of the 3D-printed glenohumeral fusion prosthesis is provided by screws inserted into the neck of the scapula, a metal plate fixed on the spine of the scapula, and the frustum of a cone structure embedded in the scapular neck. Long-term stability is provided by bone ingrowth from the osteotomy interface of the glenoid cavity to the contact interface of the 3D-printed porous structure of the prosthesis (as indicated by the red arrows in Figures 1A, B). That is, the surface of the frustum of a cone structure embedded in the scapular neck and the interface between the prosthesis and osteotomy in the glenoid cavity are made into a 3- to 5-mm-thick, porous structure (trabecular bone structure) by 3D printing technology so that the bone can quickly grow into the interface and achieve biological fusion. The 3D-printed glenohumeral fusion prosthesis was fabricated by Arcam Q10 PLUS (Arcam Inc., Germany) using high-energy electron beam melting (EBM) technology. The printing material was titanium alloy (Ti-6Al-4V). The porosity of the porous structure was 60%–85%, and the pore size was 300 µm-800 µm, which had been proven to achieve good integration of the implant and bone interface (Wang et al., 2016). Porous structures of titanium alloys made by 3D printing technology have been proven to have properties precisely supporting bone fusion (Ji et al., 2020).

The position of shoulder fusion is generally at 20° of abduction, 30° of forward flexion, and 40° of internal rotation after reconstruction (Wang et al., 2011).

### 2.2 Finite element analysis verification

Computed tomography (CT) (united-imaging CT960+, United Imaging Healthcare Co. Ltd., China) was used to scan the shoulder joint and upper limb of a healthy adult male at a slice thickness of 1 mm. The functions of CT were set to 320 rows and 640 layers, and the scanning parameters were set to standard tube voltage 70–140 KV, tube current 10–833 ma, and the MAC algorithm was used for images reconstruction. The images obtained were used for 3D model reconstruction. The use of the personal imaging data was approved by the hospital’s ethics committee and agreed to by the volunteer. The scanning data were imported into the three-dimensional (3D) reconstruction software Mimics (Materialise NV, Leuven, Belgium) in DICOM format. After mask processing, the data were exported in STL format. The STL files were subsequently transferred to Geomagic Design software (Research Triangle Park, Durham, NC, United States) for reconstruction by reverse engineering techniques. SolidWorks software (Waltham, MA, United States) was used for 3D solid modeling.

Three reconstruction models were created, all of which simulated reconstruction of the bone defect after a 15-cm osteotomy in the proximal humerus. Model 1 represented traditional glenohumeral arthrodesis with an intercalary allograft and a vascularized fibular graft (Figure 1D). Model 2 represented the 3D-printed glenohumeral fusion prosthesis (Figure 1E), and Model 3 represented the 3D-printed glenohumeral fusion prosthesis with a metal plate fixed on the spine of the scapula (lateral plate fixation) (Figure 1F).

The assembled solid model was imported into ANSYS Workbench (Ansys, Inc., Canonsburg, PA, United States), where Boolean operations were carried out. The material of the prosthesis and metal plate were set to titanium alloy (Ti-6Al-4 V), and the material properties are shown in Supplementary Table S1 (Yang et al., 2013).

The friction setting was established between individual components according to the actual situation. Contact binding was created between the screws, metal plate and the prosthesis, and the fretting was ignored. Friction contact was used on the interface between bone and prosthesis, the friction coefficient was 0.3 (Ji et al., 2010). The mesh convergence test was carried out for the mesh size, and the calculation analysis was carried out for the five dimensional finite element models with mesh sizes of 2, 3, 4, 5, and 6 mm. Compared with the maximum equivalent stress of shoulder prosthesis, when the mesh size was 2 mm and 3 mm, the maximum stress value was less than 2%, which met the requirement of 5% reported in the literature. Considering the time efficiency of calculation, 3 mm was chosen as the final mesh size in this study. The grid was divided freely, with solid 187 as the solid unit and 3 mm as the unit size (Zhou et al., 2020) (Figure 1G). Model 1 consisted of 1,26,780 elements and 2,05,042 nodes. Model 2 consisted of 1,42,213 elements and 2,17,636 nodes. Model 3 consisted of 1,52,693 elements and 2,38,627 nodes. The scapula was bound and fixed, and two loading modes were applied to the distal end of the humerus. The first loading mode consisted of an axial load of 700 N along the humeral axis, which was equivalent to the pressure on the glenohumeral joint during arm elevation (Yang et al., 2013) (Figure 1H, Model 1 is taken as an example to illustrate two loading modes). The loading mode consisted of a vertical downward load of 42.532 N (Codsi and Iannotti, 2008), which was equivalent to the weight of the upper limb (Anglin et al., 2000) (Figure 1I). The displacements and von Mises stresses of the three models were observed.

### 2.3 Biomechanical experiment verification

The results of finite element analysis proved that the 3D-printed glenohumeral fusion prosthesis exhibited better mechanical properties than traditional glenohumeral arthrodesis. On the premise that the fusion prosthesis was superior to the traditional method, the finite element analysis also showed that adding metal plate fixation could better stabilize the fusion prosthesis and improve its mechanical properties. To further prove the second conclusion of the finite element analysis, we designed a biomechanical experiment to verify whether the novel fusion prosthesis actually required additional lateral plate fixation.

Biomimetic bones (sawbones) of the shoulder joint were used to construct biomechanical experimental models for simulating proximal humerus bone defect reconstruction after proximal humerus tumor resection with the 3D-printed glenohumeral fusion prosthesis (Figure 1C). Biomimetic bones (sawbones) were produced by the Shanghai Innuo Industrial Co. Ltd. (Shanghai, China) based on the CT data of the shoulder and upper arm used in the finite element analysis as described above.

The preparation process of the models was as follows. A grinding drill was used to create a groove in the center of the glenoid of the biomimetic scapula that just accommodated the frustum of a cone structure of the prosthesis, so that it had a certain pullout friction after embedding and was close to the bone surface exactly. The position of the prosthesis was adjusted so that the prosthesis was at 20° of abduction, 30° of forward flexion, and 40° of internal rotation after reconstruction. The orthopedic electric drill with a 3.5 mm drill bit was used to drill through the screw hole of the main part of the prosthesis. Four screws were screwed into the hole to fix it to the glenoid. Along with the lateral nail path of the main part of the prosthesis, the reconstruction metal plate was placed in the middle of the scapular spine as an appropriate position and then screwed into the screw to fix the prosthesis on the scapular spine.

The models were classified into two groups: the first group included models reconstructed using the 3D-printed glenohumeral fusion prosthesis with a lateral metal plate fixed on the spine of the scapula (with-plate group) (Figures 5A, C); the other group included models reconstructed using the 3D-printed glenohumeral fusion prosthesis only (without-plate group) (Figures 5B, D). There were 6 samples in each group. A uniaxial pressure test was performed using a pressure tester (Electronic universal pressure testing machine, Shenzhen Sansheng Technology Co., Ltd., Shenzhen, China). Vertical downward pressure was applied to the prosthetic along the axis of the humerus. The loading rate was set at 0.05 mm/s, and the maximum pressure of the load was 700 N (as in the first loading mode in the finite element analysis). Moreover, the force–displacement curve of the 3D-printed glenohumeral fusion prosthesis was evaluated using a noncontact full-field dynamic strain measurement system (VIC-3D system, Correlated Solutions, Irmo, SC, United States) (system accuracy, ± 0.1 μm) (Zhou et al., 2020).

## 3 Results

### 3.1 Results of the finite element analysis

#### 3.1.1 Displacement under different loading modes

**FIGURE 2:**
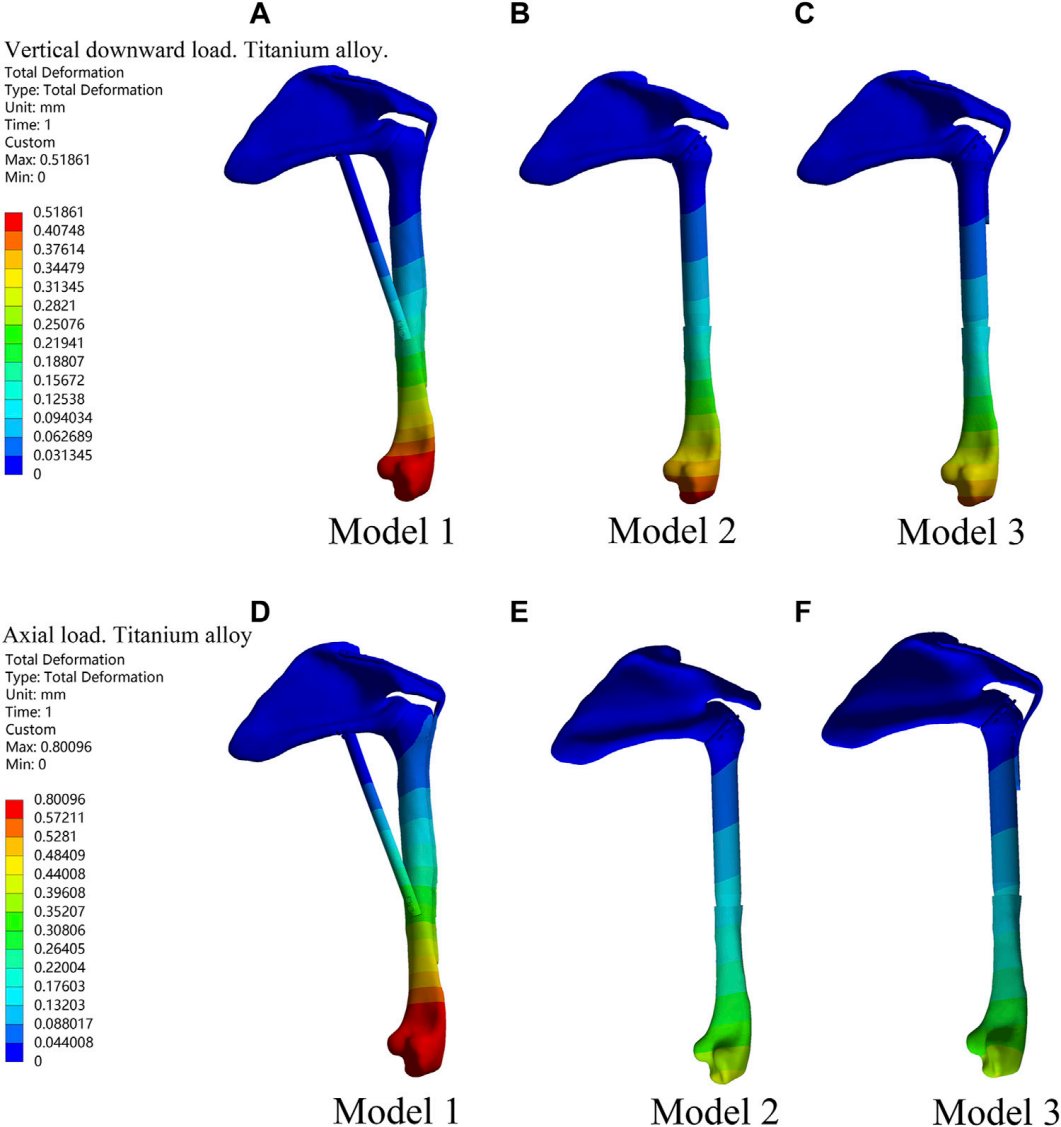
Displacement diagrams of three reconstruction models under the two different loads in the finite element analysis. **(A–C)** Displacement diagrams of three reconstruction models under the vertical downward load. **(D–F)** Displacement diagrams of three reconstruction models under the axial load.

Under the two different loading modes, the displacements of the three models were compared. The results showed the smallest displacement for Model 3 among the three models (Figure 2; Table 1). In terms of displacement, the overall stability of the novel fusion prosthesis was greater than that of traditional glenohumeral joint arthrodesis.

**TABLE 1:** Displacements of the three models under different loadings in the finite element analysis.

Models	Displacements (mm)	
	Under vertical downward loading	Under axial loading
Model 1	0.8010	0.5186
Model 2	0.4189	0.4335
Model 3	0.3981	0.3956

#### 3.1.2 Von mises stress of the three models

**FIGURE 3:**
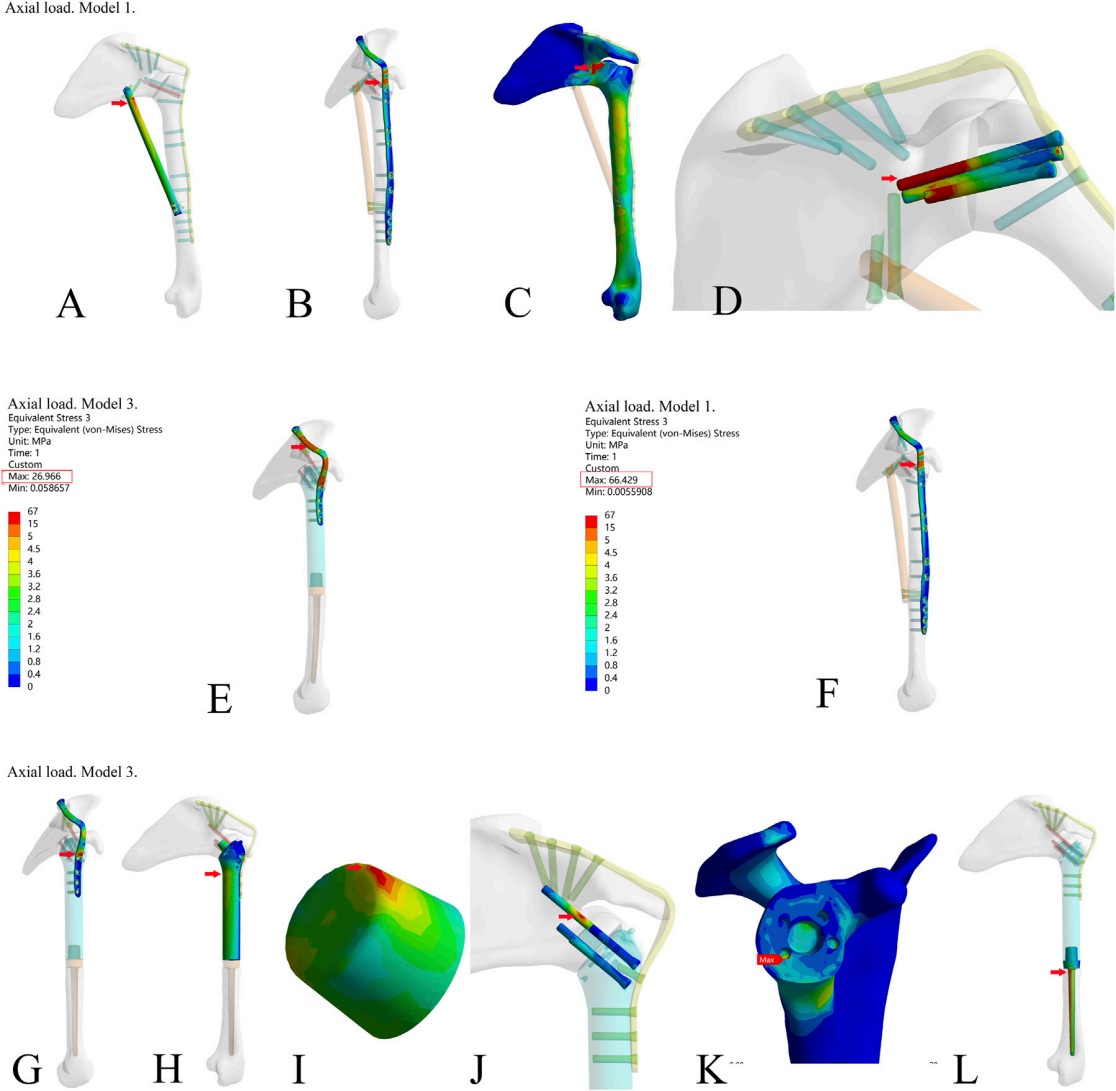
The stress of each component in the three reconstruction models under the axial load in the finite element analysis. **(A–D)** The stress of fibula, metal plate, bone, and screws (through the glenoid articular surface) in model 1; **(E,F)** Stress of metal plate in Model 3 and Model 1; **(G–L)** Stress of metal plate, prosthesis main body, the frustum of a cone, screws (through the glenoid articular surface), bone, and prosthesis stem in Model 3. (The frustum of a cone is a part of the prosthesis’s main body. The frustum of a cone and the prosthesis’s main body is an integrated structure. For the sake of observation, the frustum of a cone is shown separately.)

To further compare the mechanical properties of the three reconstruction methods, we investigated the von Mises stress of each component (Table 2). The results showed that there were stress concentration points in Model 1 under the axial load (Figures 3A–D). Under the two different loading modes, the stress in the metal plate in Model 3 was much lower than that in Model 1, and the stress was dispersed (Figures 3E, F). Under the axial load, the maximum stress in the metal plate in Model 1 was approximately 1.5 times that in Model 3 (66.429 N vs. 26.96 N), and the stress in the metal plate in Model 3 was dispersed into multiple parts. Under the vertical downward load, the maximum stress in the metal plate in Model 1 was more than three times that in Model 3 (58.234 N vs. 16.9 N). The stress distribution in all parts of Model 3 was relatively rational, and there were no points of stress concentration (Figures 3G–L). Therefore, the design of the novel fusion prosthesis was rational. There were no obvious points of stress concentration in the internal part, the stable structure (metal plate or screws) bore most of the stress, and the force was well distributed.

**TABLE 2:** Von Mises stress of the three models under different loadings in the finite element analysis.

Parameter	Component	Loading modes	Models		
			Model 1	Model 2	Model 3
Von Mises stress (Mpa)	Metal plate	Loading 1	66.429	NA	26.966
		Loading 2	58.234	NA	16.900
	Screws	Loading 1	25.339	33.395	26.908
		Loading 2	8.087	18.023	15.229
	Bone	Loading 1	20.680	30.850	28.677
		Loading 2	6.584	9.031	7.248
	The frustum of a cone	Loading 1	NA	11.590	9.931
		Loading 2	NA	5.647	5.227
	Prosthesis main body	Loading 1	NA	15.896	13.625
		Loading 2	NA	14.929	13.386
	Prosthesis stem	Loading 1	NA	14.798	14.793
		Loading 2	NA	11.056	10.994
	Fibular graft	Loading 1	12.101	NA	NA
		Loading 2	9.583	NA	NA

1) Loading 1: Under axial loading. 2) Loading 2: Under vertical downward loading. 3) NA: not available.

**FIGURE 4:**
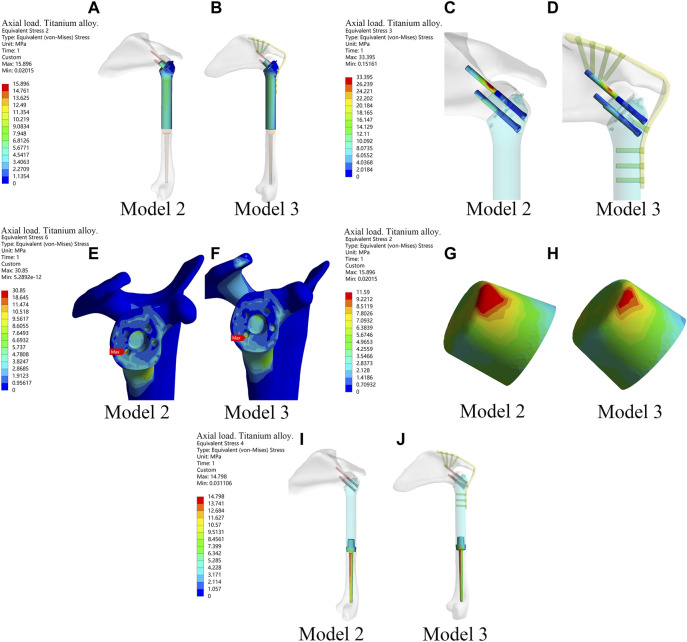
The stress of each component of the new prosthesis under the axial load with and without metal plates and screws fixed on the scapula in the finite element analysis. **(A,B)** The stress of prosthesis main body in Model 2 and Model 3. **(C,D)** Stress of screws in Model 2 and Model 3. **(E,F)** Stress of bone in Model 2 and Model 3. **(G,H)** Stress of the frustum of a cone in Model 2 and Model 3. **(I,J)** Stress of prosthetic stem in Model 2 and Model 3.

The results above showed that the novel fusion prosthesis exhibited better mechanical properties than traditional glenohumeral joint arthrodesis. Furthermore, we compared the forces of each component of the novel prosthesis under the two different loads with and without metal plates and screws fixed on the scapula. The results showed that the stress in each part was lower in Model 3 than in Model 2 under the two different loads (Figure 4). Therefore, adding metal plate fixation could better stabilize the fusion prosthesis and improve its mechanical properties.

### 3.2 Results of the biomechanical experiment

#### 3.2.1 Displacement

**FIGURE 5:**
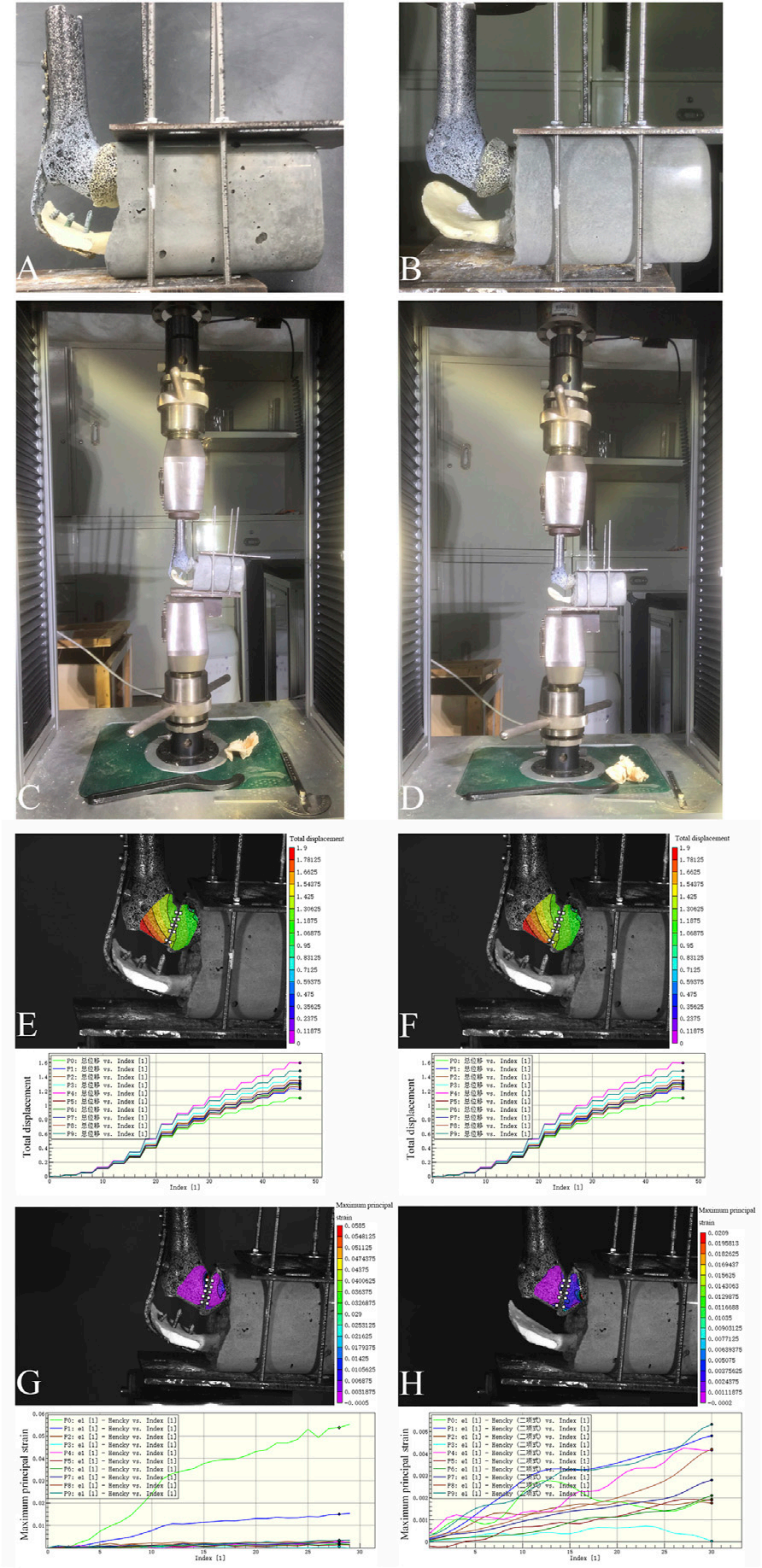
Two reconstruction models in the biomechanical experiment verification, and the total displacement of the prosthesis and the maximum principal strain of the glenoid cavity at an axial downward pressure of 700 N in the biomechanical experiment. **(A,C)** The reconstruction model using the 3D-printed glenohumeral fusion prosthesis with a lateral metal plate fixed on the spine of the scapula (with-plate group); **(B,D)** The reconstruction model using the 3D-printed glenohumeral fusion prosthesis only (without-plate group). **(E)** The total displacement of the prosthesis in the with-plate group. **(F)** The total displacement of the prosthesis in the without-plate group. **(G)** The maximum principal strain of the glenoid cavity in the with-plate group. **(H)** The maximum principal strain of the glenoid cavity in the without-plate group.

When the axial downward pressure was 700 N, the difference in the average total displacement between the with-plate group and the without-plate group was 1.1491 mm, which was statistically significant (*P* = 0.012) (Table 3). Figure 5E, F show the total displacement of the 3D-printed glenohumeral fusion prosthesis relative to the scapula (Supplementary Figure S1 shows the displacement on three axes.). Without lateral plate fixation, the total displacement of the prosthesis doubled.

**TABLE 3:** Comparison of total displacement and maximum principal strain between the with-plate group and the without-plate group at axial downward pressure of 700 N in the biomechanical experiment verificationa

Parameters	The with-plate group	The without-plate group	*p* value
Total displacement (mm)	1.1526 ± 0.1178	2.3017 ± 0.6063	0.012
Maximum principal strain	0.0013 ± 0.0003	0.0021 ± 0.0007	0.047

Studies have shown that micromotion of less than 150 μm is a necessary condition for bone growth and biological fusion, which prolongs the efficacy of implants (Pilliar et al., 1986; Engh et al., 1992; Jasty et al., 1997; Kienapfel et al., 1999). We used a total displacement of 150 μm as the critical value to observe the loading pressure on the prosthesis. For the 6 samples in the with-plate group, the total displacement was greater than 150 μm after the force was greater than 150 N (15 kg). For the 6 samples in the without-plate group, the total displacement was greater than 150 μm after the force was greater than 50 N (5 kg).

#### 3.2.2 Maximum principal strain

Under an axial downward pressure of 700 N, the maximum principal strain in the without-plate group was obviously greater, and the difference in the average maximum principal strain between the two groups was statistically significant (*P* = 0.047) (Table 3). However, the difference was not only numerical; analysis of the strain distribution also showed that the strain distribution in the glenoid in the with-plate group was relatively uniform and dispersed, while that in the without-plate group was relatively chaotic and locally concentrated (Figure 5G, H). Therefore, adding lateral plate fixation could reduce and disperse the strain on the glenoid.

## 4 Discussion

Each of the available methods for reconstruction after the resection of proximal humerus bone tumors has disadvantages, and the optimal reconstruction method is still uncertain. Although autologous bone graft reconstruction provides a good rate of bone fusion, the method still has some disadvantages in cases of large bone defects, such as limited sources of available autologous bone and insufficient strength of bone grafts, which may result in fractures (Li et al., 2012; Kubo et al., 2016; Barbier et al., 2017; Shammas et al., 2017; Stevenson et al., 2018). Allografts are used in several reconstruction methods, including osteoarticular allografting, the application of allograft-prosthesis composites, and arthrodesis with an intercalary allograft and vascularized fibular graft. Although allografts are ideal materials for bone reconstruction, they may lead to biological and biomechanical complications, such as delayed union or nonunion of the graft, bone resorption, fracture, cartilage degeneration, and joint instability after reconstruction (Teunis et al., 2014; Nota et al., 2018; El Beaino et al., 2019; Yao et al., 2020). Artificial prosthesis placement includes the placement of anatomical proximal humerus prostheses and reverse humerus prostheses. The combination of anatomical proximal humerus prostheses with the scapular glenoid and soft tissue makes it difficult to ensure the stability of the joint by reinforcement with mesh patches and anchors, which may cause shoulder dislocation or subluxation and shoulder instability, especially in patients treated with extraarticular tumor resection (Van De Sande et al., 2011; Liu et al., 2014; Teunis et al., 2014; Tang et al., 2015; Yang et al., 2021). A reverse humerus prosthesis may result in postoperative shoulder joint dysfunction due to failure to preserve the deltoid or axillary nerve; only when the deltoid or axillary nerve is intact can a reverse humerus prosthesis exhibit relatively good clinical efficacy (Arne. et al., 2014; Guven et al., 2016; Lazerges et al., 2017). After Malawer type IB resection of the proximal humerus, arthrodesis with an allograft and vascularized fibular graft is recommended for reconstruction in younger, more active patients (O’Connor et al., 1996; Wang et al., 2011; Bilgin, 2012; Mimata et al., 2015). However, the drawbacks of this method include the long operation time, extreme trauma, long time required for fusion between the allograft and host bone, rejection reactions, bone resorption, and bone nonunion, which may lead to additional related complications and ultimately cause reconstruction failure (Fuchs et al., 2005; Wang et al., 2011; Bilgin, 2012; Wieser et al., 2013; Padiolleau et al., 2014; Mimata et al., 2015). Therefore, the clinical application of this surgical method has gradually decreased. The so-called glenohumeral fusion prosthesis (O’Connor et al., 1996) made by traditional techniques relies on only a few screws to achieve stability in the initial stage of reconstruction but cannot provide long-term biological stability via integration between the bone and prosthesis. As a result, in the long term, the failure rate of these prostheses is very high.

To avoid the disadvantages of the above glenohumeral nonfusion reconstruction methods and the traditional so-called glenohumeral joint fusion reconstruction method, we innovatively designed a novel 3D-printed glenohumeral fusion prosthesis, which provides an important reference for reconstruction after malignant proximal humerus tumor resection. The design concept of the glenohumeral fusion prosthesis is that after shoulder joint fusion is achieved through the prosthesis, the range of motion of the shoulder joint can be partially compensated for by movement of the scapula. In addition, according to the theoretical basis of traditional glenohumeral joint arthrodesis, the glenohumeral joint fusion prosthesis based on this technology could stabilize the shoulder joint, which is an important prerequisite for the shoulder joint painless and weight-bearing functions after surgery. In this research, we demonstrated the feasibility and safety of the 3D-printed glenohumeral fusion prosthesis through finite element analysis and biomechanical assessments in preclinical experiments, which laid a solid foundation for clinical research of the prosthesis.

Biazzo et al. combined their single-institution experience and literature to describe scapular reconstruction after resection of bone tumors. After total or partial scapulectomy there are 3 options for reconstruction: humeral suspension (flail shoulder), total endoprosthesis, and massive bone allograft (Biazzo et al., 2018). Salunke et al.’s single-center retrospective cohort found that the reconstruction with polypropylene mesh had better functional outcomes and emotional acceptance as compared to the non-reconstructive group in patients with total scapular resection surgery. In addition, the findings of the systematic review suggest that patients treated by reconstruction with polypropylene mesh and the non-reconstructive group as compared to scapular prosthesis had limited shoulder movements (Salunke et al., 2024). In theory, patients who involve the removal of part of the scapula (glenoid) are also suitable for reconstruction with this novel prosthesis this study reported. Soltanmohammadi et al. performed the structural analysis of hollow versus solid-bearing shoulder implants of proximal humeri with different bone qualities. The result showed that the hollow stems maintained adequate strength and using even thinner walls may further reduce stress shielding (Soltanmohammadi et al., 2022). Therefore, we can improve the biomechanical environment of implants through structural design.

The finite element analysis results indicated that the mechanical properties of the novel glenohumeral fusion prosthesis were better than those of traditional glenohumeral joint arthrodesis. Model 1 (traditional glenohumeral joint arthrodesis) had points of stress concentration, which might be prone to rupture. Some clinical studies have reported the rupture of metal plates (Wieser et al., 2013), and other studies have even used double metal plates for reconstruction as a result (Bilgin, 2012). The stress in each part of Model 3 was lower than that in Model 2 under the two different loads, indicating that adding plate fixation could stabilize the glenohumeral fusion prosthesis and improve its mechanical properties. The stress was distributed in all parts of Model 3, which indicated that the design of the glenohumeral fusion prosthesis was rational. There were no points of obvious stress concentration in the internal part, the stable structure (metal plate or screws) bore most of the stress, and the force was well distributed. It should be noted that the design of the frustum of the cone in the novel prosthesis could greatly resist the vertical shear stress of the articular surface. Together with the screws inserted into the neck of the scapula through the body of the prosthesis, the components maximized the initial stability and provided a mechanical environment with less fretting for bone ingrowth. According to the results of finite element analysis, it could be seen that there is no stress concentration in the bone around the prosthesis, and the stress is relatively uniform (Figures 3K, 4F). Moreover, in the mechanical experiments, analysis of the strain distribution showed that the strain distribution in the glenoid in the with-plate group was relatively uniform and dispersed (Figures 5G, H). Therefore, the stress-shielding effect of the prosthesis on the surrounding bone is relatively small.

Based on the finite element analysis, the biomechanical experiment simulated the clinical application scenario and allowed rehearsal of the surgical reconstruction and installation steps, which showed the strong feasibility of the novel prosthesis. The biomechanical experiment confirmed the excellent initial stability of the glenohumeral fusion prosthesis and confirmed that the addition of a lateral plate further inhibited fretting, reduced the maximum principal strain, and dispersed the strain on the glenoid side, thereby creating a stable environment for bone ingrowth (Pilliar et al., 1986; Engh et al., 1992; Jasty et al., 1997; Kienapfel et al., 1999). Our biomechanical experiment showed that the micromotion of the construct was less than 150 μm, which is a necessary condition for bone ingrowth and biological fusion and allows implants to remain effective for a long time.

The limitations of this study are as follows: First, in the mechanical analysis, only two kinds of forces on the upper limb were simulated; Second, the biomechanical study provides preclinical evidence for the feasibility and safety of the novel prosthesis, but further clinical studies are needed to verify its clinical efficacy.

## 5 Conclusion

The design of the 3D-printed glenohumeral fusion prosthesis was rational, and its stability and mechanical properties were better than those of traditional glenohumeral joint arthrodesis. Biomechanical verification demonstrated the feasibility and safety of this prosthesis, indicating its potential for proximal humerus bone defect reconstruction after proximal humerus tumor resection.

## Data Availability

The original contributions presented in the study are included in the article/Supplementary Material, further inquiries can be directed to the corresponding author.

## References

[B1] Abdeen A. Hoang B. H. Athanasian E. A. Morris C. D. Boland P. J. Healey J. H. (2009). Allograft-prosthesis composite reconstruction of the proximal part of the humerus. Functional outcome and survivorship. J. Bone Jt. Surg. - Ser. A 91, 2406–2415. 10.2106/JBJS.H.00815 19797576

[B2] Anglin C. Wyss U. P. Pichora D. R. (2000). Glenohumeral contact forces. Proc. Inst. Mech. Eng. Part H. J. Eng. Med. 214, 637–644. 10.1243/0954411001535660 11201411

[B3] Arne S. Marcel H. Georg G. Helmut A. Markus N. Wiebke G. (2014). Improvement of the shoulder function after large segment resection of the proximal humerus with the use of an inverse tumour prosthesis. Int. Orthop. 39, 355–361. 10.1007/s00264-014-2560-2 25326856

[B4] Barbier D. De Billy B. Gicquel P. Bourelle S. Journeau P. (2017). Is the clavicula pro humero technique of value for reconstruction after resection of the proximal humerus in children? Clin. Orthop. Relat. Res. 475, 2550–2561. 10.1007/s11999-017-5438-y 28699149 PMC5599409

[B5] Biazzo A. De Paolis M. Donati D. M. (2018). Scapular reconstructions after resection for bone tumors: a single-institution experience and review of the literature. Acta Biomed. 89, 415–422. 10.23750/abm.v89i3.5655 30333470 PMC6502122

[B6] Bilgin S. S. (2012). Reconstruction of proximal humeral defects with shoulder arthrodesis using free vascularized fibular graft. J. Bone Jt. Surg. - Ser. A 94, e94. 10.2106/JBJS.J.01823 22760395

[B7] Codsi M. J. Iannotti J. P. (2008). The effect of screw position on the initial fixation of a reverse total shoulder prosthesis in a glenoid with a cavitary bone defect. J. Shoulder Elb. Surg. 17, 479–486. 10.1016/j.jse.2007.09.002 18282725

[B8] El Beaino M. Liu J. Lewis V. O. Lin P. P. (2019). Do early results of proximal humeral allograft-prosthetic composite reconstructions persist at 5-year followup? Clin. Orthop. Relat. Res. 477, 758–765. 10.1097/CORR.0000000000000354 30811366 PMC6437392

[B9] Engh C. A. O’Connor D. Jasty M. McGovern T. F. Bobyn J. D. Harris W. H. (1992). Quantification of implant micromotion, strain shielding, and bone resorption with porous-coated anatomic medullary locking femoral prostheses. Clin. Orthop. Relat. Res. 285, 13–29. 10.1097/00003086-199212000-00005 1446429

[B10] Fuchs B. O’Connor M. I. Padgett D. J. Kaufman K. R. Sim F. H. (2005). Arthrodesis of the shoulder after tumor resection. Clin. Orthop. Relat. Res., 202–207. 10.1097/01.blo.0000162997.31976.15 15995442

[B11] Guven M. F. Aslan L. Botanlioglu H. Kaynak G. Kesmezacar H. Babacan M. (2016). Functional outcome of reverse shoulder tumor prosthesis in the treatment of proximal humerus tumors. J. Shoulder Elb. Surg. 25, e1–e6. 10.1016/j.jse.2015.06.012 26234664

[B12] Jasty M. Bragdon C. Burke D. O’Connor D. Lowenstein J. Harris W. H. (1997). *In vivo* skeletal responses to porous-surfaced implants subjected to small induced motions. J. Bone Jt. Surg. - Ser. A 79, 707–714. 10.2106/00004623-199705000-00010 9160943

[B13] Ji T. Guo W. Tang X. D. Yang Y. (2010). Reconstruction of type II+III pelvic resection with a modular hemipelvic endoprosthesis: a finite element analysis study. Orthop. Surg. 2, 272–277. 10.1111/j.1757-7861.2010.00099.x 22009962 PMC6583553

[B14] Ji T. Yang Y. Tang X. Liang H. Yan T. Yang R. (2020). 3D-Printed modular hemipelvic endoprosthetic reconstruction following periacetabular tumor resection: early results of 80 consecutive cases. J. Bone Jt. Surg. Am. 102, 1530–1541. 10.2106/JBJS.19.01437 32427766

[B15] Kienapfel H. Sprey C. Wilke A. Griss P. (1999). Implant fixation by bone ingrowth. J. Arthroplasty. 14, 355–368. 10.1016/S0883-5403(99)90063-3 10220191

[B16] Kubo T. Furuta T. Ochi M. (2016). More than 20-year follow-up after vascularised fibula head graft for oncological shoulder joint reconstruction. Anticancer Res. 36, 301–305. 26722057

[B17] Lazerges C. Dagneaux L. Degeorge B. Tardy N. Coulet B. Chammas M. (2017). Composite reverse shoulder arthroplasty can provide good function and quality of life in cases of malignant tumour of the proximal humerus. Int. Orthop. 41, 2619–2625. 10.1007/s00264-017-3538-7 28646420

[B18] Li J. Wang Z. Guo Z. Wu Y. Chen G. Pei G. (2012). Precise resection and biological reconstruction for patients with bone sarcomas in the proximal humerus. J. Reconstr. Microsurg. 28, 419–425. 10.1055/s-0032-1315766 22711209

[B19] Liu T. Zhang Q. Guo X. Zhang X. Li Z. Li X. (2014). Treatment and outcome of malignant bone tumors of the proximal humerus: biological versus endoprosthetic reconstruction. BMC Musculoskelet. Disord. 15, 69. 10.1186/1471-2474-15-69 24607200 PMC3975708

[B20] Mimata Y. Nishida J. Sato K. Suzuki Y. Doita M. (2015). Glenohumeral arthrodesis for malignant tumor of the shoulder girdle. J. Shoulder Elb. Surg. 24, 174–178. 10.1016/j.jse.2014.05.023 25174936

[B21] Nota S. Teunis T. Kortlever J. Ferrone M. Ready J. Gebhardt M. (2018). Functional outcomes and complications after oncologic reconstruction of the proximal humerus. J. Am. Acad. Orthop. Surg. 26, 403–409. 10.5435/JAAOS-D-16-00551 29762195

[B22] O’Connor M. I. Sim F. H. Chao E. Y. S. (1996). Limb salvage for neoplasms of the shoulder girdle: intermediate reconstructive and functional results. J. Bone Jt. Surg. - Ser. A 78, 1872–1888. 10.2106/00004623-199612000-00011 8986665

[B23] Padiolleau G. Marchand J. B. Odri G. A. Hamel A. Gouin F. (2014). Scapulo-humeral arthrodesis using a pedicled scapular pillar graft following resection of the proximal humerus. Orthop. Traumatol. Surg. Res. 100, 181–185. 10.1016/j.otsr.2013.09.012 24507409

[B24] Pilliar R. M. Lee J. M. Maniatopoulos C. (1986). Observations on the effect of movement on bone ingrowth into porous-surfaced implants. Clin. Orthop. Relat. Res. 208, 108–113. 10.1097/00003086-198607000-00023 3720113

[B25] Salunke A. A. Nandy K. Kamani M. Parmar R. Bharwani N. Pathak S. (2024). Is polypropylene mesh reconstruction functionally superior to non reconstructive group following total scapular resection? A retrospective analysis of 16 patients and a systematic review of the literature. J. Orthop. 52, 37–48. 10.1016/j.jor.2024.02.019 38404696 PMC10891286

[B26] Shammas R. L. Avashia Y. J. Farjat A. E. Catanzano A. A. Levin L. S. Eward W. C. (2017). Vascularized fibula-based physis transfer: a follow-up study of longitudinal bone growth and complications. Plast. Reconstr. Surg. - Glob. Open 5, e1352. 10.1097/GOX.0000000000001352 28607872 PMC5459655

[B27] Sirveaux F. (2019). Reconstruction techniques after proximal humerus tumour resection. Orthop. Traumatol. Surg. Res. 105, S153–S164. 10.1016/j.otsr.2018.04.024 29958931

[B28] Soltanmohammadi P. Tavakoli A. Langohr G. D. G. Athwal G. S. Willing R. (2022). Structural analysis of hollow versus solid-stemmed shoulder implants of proximal humeri with different bone qualities. J. Orthop. Res. 40, 674–684. 10.1002/jor.25076 33969537

[B29] Stevenson J. D. Doxey R. Abudu A. Parry M. Evans S. Peart F. (2018). Vascularized fibular epiphyseal transfer for proximal humeral reconstruction in children with a primary sarcoma of bone. Bone Jt. J. 100-B, 535–541. 10.1302/0301-620X.100B4.BJJ-2017-0830.R1 29629581

[B30] Tang X. Guo W. Yang R. Tang S. Ji T. (2015). Synthetic mesh improves shoulder function after intraarticular resection and prosthetic replacement of proximal humerus. Clin. Orthop. Relat. Res. 473, 1464–1471. 10.1007/s11999-015-4139-7 25604875 PMC4353552

[B31] Teunis T. Nota S. P. F. T. Hornicek F. J. Schwab J. H. Lozano-Calderón S. A. (2014). Outcome after reconstruction of the proximal humerus for tumor resection: a systematic review. Clin. Orthop. Relat. Res. 472, 2245–2253. 10.1007/s11999-014-3474-4 24469551 PMC4048415

[B32] Van De Sande M. A. J. Sander Dijkstra P. D. Taminiau A. H. M. (2011). Proximal humerus reconstruction after tumour resection: biological versus endoprosthetic reconstruction. Int. Orthop. 35, 1375–1380. 10.1007/s00264-010-1152-z 21085956 PMC3167452

[B33] Wang B. Wu Q. Liu J. Yang S. Shao Z. (2015). Endoprosthetic reconstruction of the proximal humerus after tumour resection with polypropylene mesh. Int. Orthop. 39, 501–506. 10.1007/s00264-014-2597-2 25416123

[B34] Wang J. Shen J. Dickinson I. C. (2011). Functional outcome of arthrodesis with a vascularized fibular graft and a rotational latissimus dorsi flap after proximal humerus sarcoma resection. Ann. Surg. Oncol. 18, 1852–1859. 10.1245/s10434-010-1443-z 21331810

[B35] Wang X. Xu S. Zhou S. Xu W. Leary M. Choong P. (2016). Topological design and additive manufacturing of porous metals for bone scaffolds and orthopaedic implants: a review. Biomaterials 83, 127–141. 10.1016/j.biomaterials.2016.01.012 26773669

[B36] Wieser K. Modaressi K. Seeli F. Fuchs B. (2013). Autologous double-barrel vascularized fibula bone graft for arthrodesis of the shoulder after tumor resection. Arch. Orthop. Trauma Surg. 133, 1219–1224. 10.1007/s00402-013-1795-5 23793479

[B37] Wittig J. C. Kellar-graney K. L. Malawer M. M. Bickels J. Meller I. (2001). Limb-sparing Surgery for high-grade sarcomas of the proximal humerus. Tech. Shoulder Elb. Surg. 2, 54–69. 10.1097/00132589-200103000-00007

[B38] Yang C. C. Lu C. L. Wu C. H. Wu J. J. Huang T. L. Chen R. (2013). Stress analysis of glenoid component in design of reverse shoulder prosthesis using finite element method. J. Shoulder Elb. Surg. 22, 932–939. 10.1016/j.jse.2012.09.001 23312816

[B39] Yang Y. Li Y. Liu W. Niu X. (2021). Mesh patch and anchors can improve clinical results of prosthetic replacement after resection of primary proximal humerus malignant tumor. Sci. Rep. 11, 734. 10.1038/s41598-020-78959-y 33436664 PMC7804124

[B40] Yao W. Cai Q. Wang J. Hou J. (2020). Mid-to long-term effects of two different biological reconstruction techniques for the treatment of humerus osteosarcoma involving caput humeri. World J. Surg. Oncol. 18, 23. 10.1186/s12957-020-1797-z 31996228 PMC6990589

[B41] Zhou L. Lin J. Huang A. Gan W. Zhai X. Sun K. (2020). Modified cannulated screw fixation in the treatment of Pauwels type III femoral neck fractures: a biomechanical study. Clin. Biomech. 74, 103–110. 10.1016/j.clinbiomech.2020.02.016 32155446

