# Supplementary material for: Design and validation of a novel 3D-printed glenohumeral fusion prosthesis for the reconstruction of proximal humerus bone defects: a biomechanical study
Source: Front Bioeng Biotechnol. 2024 Jul 8;12:1428446. doi: 10.3389/fbioe.2024.1428446 (PMC11260710; doi:10.3389/fbioe.2024.1428446)
Supplement: Supplementary file 1 [file DataSheet1.docx]

Supplementary Material

# Supplementary Figures


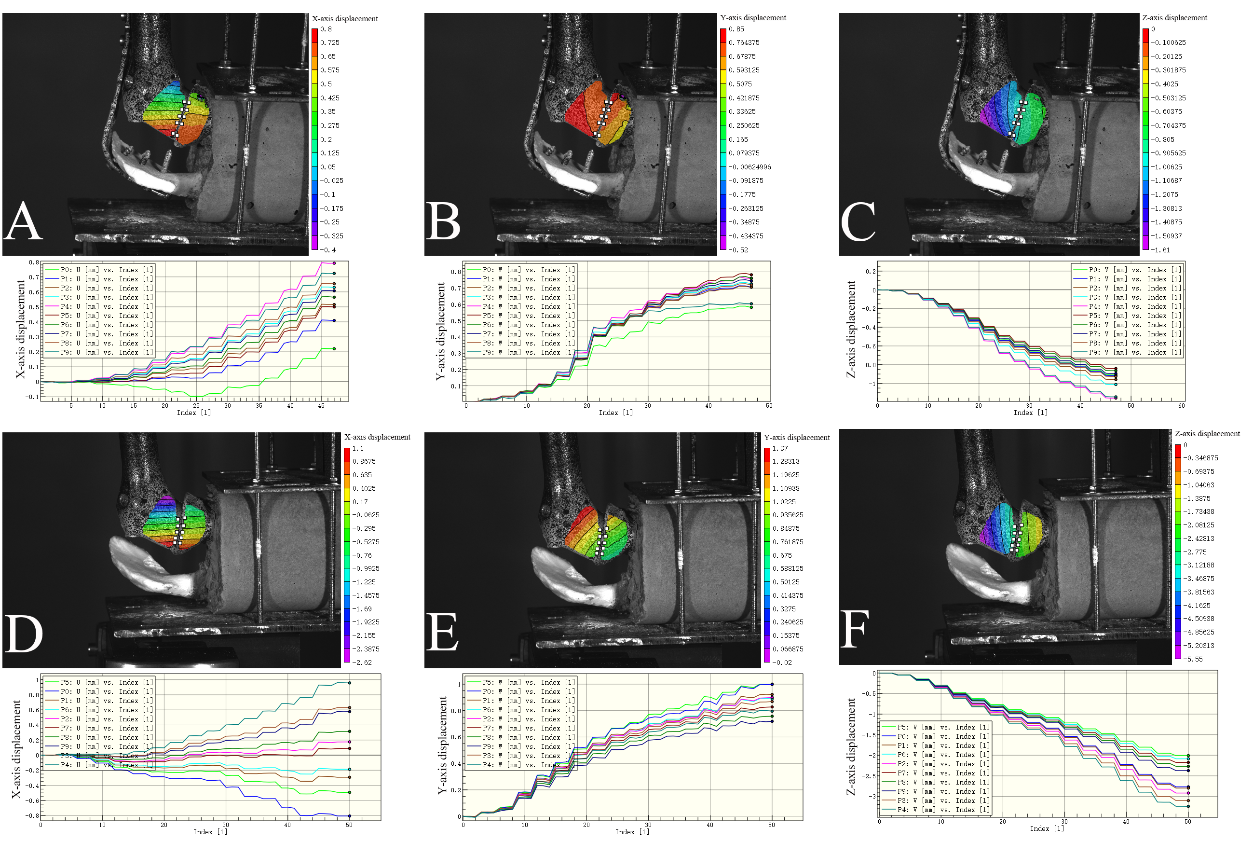


**Supplementary Figure 1.** The displacement of the prosthesis on three axes at an axial downward pressure of 700 N in the biomechanical experiment verification. (A) The displacement of the prosthesis on the X-axis in the with-plate group. (B) The displacement of the prosthesis on the Y-axis in the with-plate group. (C) The displacement of the prosthesis on the Z-axis in the with-plate group. (D)The displacement of the prosthesis on the X-axis in the without-plate group. (E) The displacement of the prosthesis on the Y-axis in the without-plate group. (F) The displacement of the prosthesis on the Z-axis in the without-plate group.

# Supplementary Tables

**Supplementary** **Table 1.** The material properties used in the finite element analysis

|  | Young's modulus (MPa) | Poisson's Ratio |
| --- | --- | --- |
| Cortical bone | 13800 | 0.3 |
| cancellous bone | 1380 | 0.3 |
| Titanium alloy | 96000 | 0.36 |
